# Serum lncRNA THRIL predicts benign and malignant pulmonary nodules and promotes the progression of pulmonary malignancies

**DOI:** 10.1186/s12885-023-11264-9

**Published:** 2023-08-15

**Authors:** Xinyu Chen, Xianji Zhu, Wenjun Yan, Luan Wang, Dongming Xue, Shouying Zhu, Jiajun Pan, Yufeng Li, Qixiang Zhao, Dong Han

**Affiliations:** 1 Department of Cardiothoracic Surgery, Xuzhou No.1 People’s Hospital, Xuzhou Municipal Hospital Affiliated with Xuzhou Medical College, 269 Daxue Road, Xuzhou, 221000 China; 2 Department of Respiratory Medicine, Shanghai Pudong Hospital, Fudan University Pudong Medical Center, Shanghai, 201399 China

**Keywords:** Solitary pulmonary nodule, THRIL, Diagnosis, Invasion, Migration

## Abstract

**Background:**

This project aimed to research the significance of THRIL in the diagnosis of benign and malignant solitary pulmonary nodules (SPNs) and to investigate the role of THRIL/miR-99a in malignant SPNs.

**Methods:**

The study groups consisted of 169 patients with SPN and 74 healthy subjects. The differences in THRIL levels were compared between the two groups and the healthy group. The receiver operating characteristic curve (ROC) was utilized to analyze the THRIL’s significance in detecting benign and malignant SPN. Pearson correlation and binary regression coefficients represented the association between THRIL and SPN. CCK-8 assay, Transwell assay, and flow cytometry were utilized to detect the regulatory effect of THRIL silencing. The interaction between THRIL, miR-99a, and IGF1R was confirmed by the double luciferase reporter gene.

**Results:**

There were differences in THRIL expression in the healthy group, benign SPN group, and malignant SPN group. High accuracy of THRIL in the diagnosis of benign SPN and malignant SPN was observed. THRIL was associated with the development of SPN. The expression of THRIL was upregulated and miR-99a was downregulated in lung cancer cells. The double luciferase report experiment confirmed the connections between THRIL/miR-99a/IGF1R. Silencing THRIL could suppress cell proliferation, migration, and invasion and promote cell apoptosis by binding miR-99a.

**Conclusion:**

The detection of THRIL in serum is useful for the assessment of malignant SPN. THRIL can regulate the expression of IGF1R through miR-99a, thereby promoting the growth of lung cancer cells and inhibiting apoptosis.

## Background

Solitary pulmonary nodules (SPNs) are circular or quasicircular lesions less than 3 cm in diameter that occur only in the entire lung, are surrounded by air-containing lung tissue and internally opaque, and do not contain atelectasis, hilar enlargement, or the performance of pleural effusion [1, 2]. With the widespread use of advanced detection equipment such as low-dose spiral CT, the detection rate of SPNs is becoming higher [3, 4]. Although some lung nodules are benign, a significant number are early-stage potential malignancies [5]. Studies have confirmed that early screening for lung cancer and early treatment of malignant SPN can reduce the mortality rate of lung cancer patients by about 20% [6, 7]. Lung cancer is a universal malignancy and a major cause of SPN-related mortality [8]. Therefore, early detection of benign and malignant SPNs is a hot and difficult issue in current clinical work.

ncRNAs are probably the most widely studied biologically active substances [9]. Several activated ncRNAs are observed in the development of SPN. MiR-500a-3p, miR-501-3p, and miR-502-3p are dysregulated in patients with SPN, and their abnormal expression is associated with the development of SPN [10]. In the initiation and development of SPN, miR-144 is lowly expressed and it may serve as a biomarker in screening the development of SPN from benignancy to malignancy [11].LncRNA is a single-stranded non-coding RNA with an average size of over 200 nucleotides [12]. An imbalance in the expression or function of lncRNAs is considered to be an important reason for the occurrence and development of lung cancers [13, 14]. Five lncRNAs, including FAM83A-AS1, have important clinical predictive significance in patients with lung adenocarcinoma [15]. LncRNA XLOC-009167 is overexpressed in lung cancer and its predictive value is identified by ROC [16]. LncRNA THRIL is becoming a study hotspot and is related to several malignant tumors. In osteosarcoma, the expression of THRIL is enhanced in patients and is correlated with the pathogenesis of this tumor, indicating its function as a biomarker in osteosarcoma monitoring [17]. In acute lung damage led by sepsis, THRIL is upregulated, reflecting THRIL may be involved in lung injury [18]. As described above, the formation and expression of THRIL may be closely related to the growth of SPNs and can be used as a biomarker for potential malignant lung nodules.

This study aimed to investigate the differences in THRIL expression between patients with benign and malignant SPNs and healthy controls without nodules and to explore its application value in the determination of benign and malignant SPNs and screening for malignant lung nodules. Additionally, the role of THRIL in the regulation of miR-99a in lung cancer and its mechanism were elucidated.

## Materials and methods

### Collection of patients and grouping

This article was approved by the Ethics Committee of Xuzhou No.1 People’s hospital and conducted in line with the principles of the Declaration of Helsinki. A total of 169 patients with SPN and 74 healthy volunteers without lung nodules were enrolled in this study from July 2019 to February 2022, including 83 patients with benign SPN and 86 patients with malignant SPN. The inclusion criteria were as follows: chest CT examination showed a single nodule in the lung with a maximum diameter of ≤ 30 mm, with a clear border and surrounded by air-containing lung tissue, which was in line with the diagnosis of SPN [19]; no abnormality in the surrounding lung tissue, no atelectasis, and no hilar abnormality; planned to undergo histopathological examination; did not receive relevant antitumor treatment before enrolling in this study; willing to cooperate with the research; no previous history of lung cancer.

Clinicopathological parameters and results of histopathological examination of SPN patients after surgical resection were shown in Table 1.

**Table 1:** Clinicopathologic parameters of SPN patients

Parameters	Benign SPN (N = 83)	Malignant SPN (N = 86)
Location, n (%)		
Left upper lobe	14 (16.9%)	28 (32.6%)
Left lower lobe	18 (21.7%)	10 (11.6%)
Right upper lobe	29 (34.9%)	31 (36.0%)
Right middle lobe	13 (15.7%)	11 (12.8%)
Right lower lobe	9 (10.8%)	6 (7.0%)
Desity, n (%)		
Solid nodules	62 (74.7%)	58 (67.4%)
Ground glass nodules	15 (18.1%)	13 (15.1%)
Partial solid nodules	6 (7.2%)	15 (17.4%)
Size, n (%)		
< 10 mm	21 (25.3%)	14 (16.3%)
10–20 mm	43 (51.8%)	40 (46.5%)
> 20 mm	19 (22.9%)	32 (37.2%)
Histopathologic examination, n (%)		
Pneumonia	21 (25.3%)	
Pulmonary tuberculosis	7 (8.4%)	
Pulmonary hamartoma	55 (66.3%)	
Lung adenocarcinoma		67 (77.9%)
Lung squamous carcinoma		15 (17.4%)
Small cell lung carcinoma		4 (4.7%)

### Cell cultivation and transfection

H1299, HCC827, A549, PC9, H526, and DMS273 lung cancer cells and immortalized lung epithelial cells (BEAS-2B) were purchased from the Cell Bank (Shanghai, China). The cells were fostered in 1640 medium (Gibco, Carlsbad, CA, USA) containing 10% fetal bovine serum, and placed in a 37℃, 5% CO_2_ cell incubator. When the cell density reached 80–90% and was in the logarithmic growth phase, 0.25% trypsin was digested and passaged. According to the instructions, Lipofectamine 3000 (Invitrogen, Carlsbad, CA, USA) was obtained to transfect plasmids and small nucleic acids. Artificial sequences of si-THRIL (Catalogue number: 4,392,420, 5 nmol), si-NC (Catalogue number: 4,390,843, 5 nmol), miR-99a (Catalogue number: 4,464,084, 5 nmol), miR-NC (Catalogue number: 4,464,076, 5 nmol) were all purchased from Thermo Fisher (Waltham, MA, USA).

### Sampling and expression identification

In the morning before surgery, 5 ml of venous blood was collected. The blood was separated at 4℃ and serum samples were obtained, and then stored in a refrigerator at -80℃. The serum samples of participants were used to extract total RNA.

The extraction method used was Trizol solution (Invitrogen, Carlsbad, CA, USA). The mRNA was transcribed using the TaqMan microRNA reverse transcription kit (Applied Biosystems, Waltham, MA, USA). The reverse transcription kit for THRIL and IGF1R was purchased from Takara (Dalian, China). The expression of THRIL, miR-99a, or IGF1R mRNA was detected using SYBR Premix Ex Taq™ II (Takara, Japan). RT-qPCR was performed on an ABI Prism 7900HT instrument (Applied Biosystems, Foster City, CA, USA). GAPDH was utilized as a standard reference for THRIL or IGF1R expression, and U6 was adopted as an internal reference for miR-99a expression. The CT values gathered from the detection system were used to calculate the expression of target miRNA. The primers used for RT-qPCR were listed as below: THRIL forward 5’-GAGTGCAGTGGCGTGATCTC-3’, reverse 5’-AAAATTAGTCAGGCATGGTGGTG-3’; GAPDH forward 5’-GACCACAGTCCATGCCATCAC-3’, reverse 5’-ACGCCTGCTTCACCACCTT-3’; IGF1R mRNA forward 5’-AAGTTCTGGTTGTCGAGGA-3’, reverse 5’-GAGCAGCTAGAAGGGAATTAC-3’; miR-99a RT primer, 5’-GTCGTATCCAGTGCAGGGTCCGAGGTATTCGCACTGGATACGACCACAAGA-3’, forward, 5’-GCCCGTCCGATCTTGTGAA-3’, reverse, 5’-GTGCAGGGTCCGAGGT-3’; U6 RT primer 5’-GTCGTATCCAGTGCAGGGTCCGAGGTATTCGCACTGGATACGACAAAATA-3’, forward 5’-TCCGATCGTGAAGCGTTC-3’, reverse 5’-GTGCAGGGTCCGAGGT-3’.

### Commercial kits for tumor markers

The serum tumor markers carcinoembryonic antigen (CEA), carbohydrate antigen 125 (CA125), neuron-specific enolase (NSE), and cytokeratin 19 fragments 21 − 1 (CYFRA21-1) were determined using the Roche immune E602 electrochemical luminescence instrument (Basel, Switzerland) in strict accordance with the instrument and reagent operating manual.

### CCK-8 assay

Cell proliferation was analyzed according to the instructions of the Cell Counting Kit-8 (CCK-8, Dojindo Laboratories, Kumamoto, Japan). Cells were inoculated into a 96-well cell culture plate and transfected. After transfection for 0, 24, 48, and 72 h, respectively, 10 μl CCK-8 reagent was added. Absorbance at 450 nm was measured after 2 h.

### Apoptosis was detected by flow cytometry

Apoptosis analysis was performed using the Annexin V-FITC/PI Apoptosis Detection Kit (Vazyme, Nanjing, China). At 48 h after cell transfection, 1 × 10^5^ cells were collected after digestion with EDT-free trypsin, washed with PBS, and resuspended in a binding buffer. Equal amounts of V-FITC and PI were added, and the apoptotic cell ratio was determined using a BD Accuri C6 Plus flow cytometer.

### Transwell method

5 × 10^2^ cells were inoculated into transwell chambers (migration assay) or transwell chambers (invasion assay) containing Matrigel matrix gel. RPMI‑1640 medium (200 μl) was added to the upper chamber. After 48 h of culture, the supernatant was discarded. The transmembrane cells in the lower chamber were fixed with 1% paraformaldehyde, and the crystal violet solution was added. The invasion and migration of cells were counted.

### Double luciferase reporter gene assay

The targeted relationship between THRIL and miR-99a was forecasted on RNAhybrid 2.12. The target of miR-99a was predicted on TargetScan, miRWalk, and miRTarBase. The targets of miR-99a were intersected using a Venn diagram. IGF1R 3’UTR fragment, THRIL sequence, and their artificial sequences with engineered mutations at binding sites of miR-99a were inserted into the pmirGLO vector (Catalog number: E1330, Promega, USA). These vectors were named WT-IGF1R, MUT-GF1R, WT-THRIL, and MUT-THRIL, respectively. After 48 h, cells were harvested and lysed to measure luciferase activity using the Glomax 20/20 luminometer (Promega, Madison, WI, USA) according to the dual luciferase reporter assay system (Promega, USA).

### Data statistics and analysis

All types of related data were analyzed by SPSS 26.0 and GraphPad 8.0, and *P* < 0.05 was considered that there were objective statistical differences between the data. One-way and two-way ANOVA were used to compare discrepancies among groups. A binary regression method was performed to estimate the correlation between clinical variables and SPN. The ROC curve was exerted to evaluate the diagnostic efficiency of THRIL. The correlations were analyzed by the Pearson correlation method.

## Results

### General clinical data analysis

The clinical data and serological indicators of the patients were collected. No significant differences in sex, age, smoking history, and family history of tumor among healthy individuals, benign nodule group, and malignant nodule group were observed (Table 2, *P* > 0.05). The result of Table 2 indicated the comparability among these three groups.

**Table 2:** Comparisons of clinical parameters in all included individuls

Indicators	Healthy group (N = 74)	Benign SPN (N = 83)	Malignant SPN (N = 86)	*P* value
Gender (male/female)	44/30	50/33	44/42	0.422
Age (year)	56.15 ± 9.04	55.04 ± 12.18	55.67 ± 10.08	0.815
Family tumor history (Yes/No)	3/71	10/73	9/77	0.187
Smoking (Yes/No)	29/45	30/53	26/60	0.478

### The elevated trend of THRIL expression with worsening SPNs

**Fig. 1:**
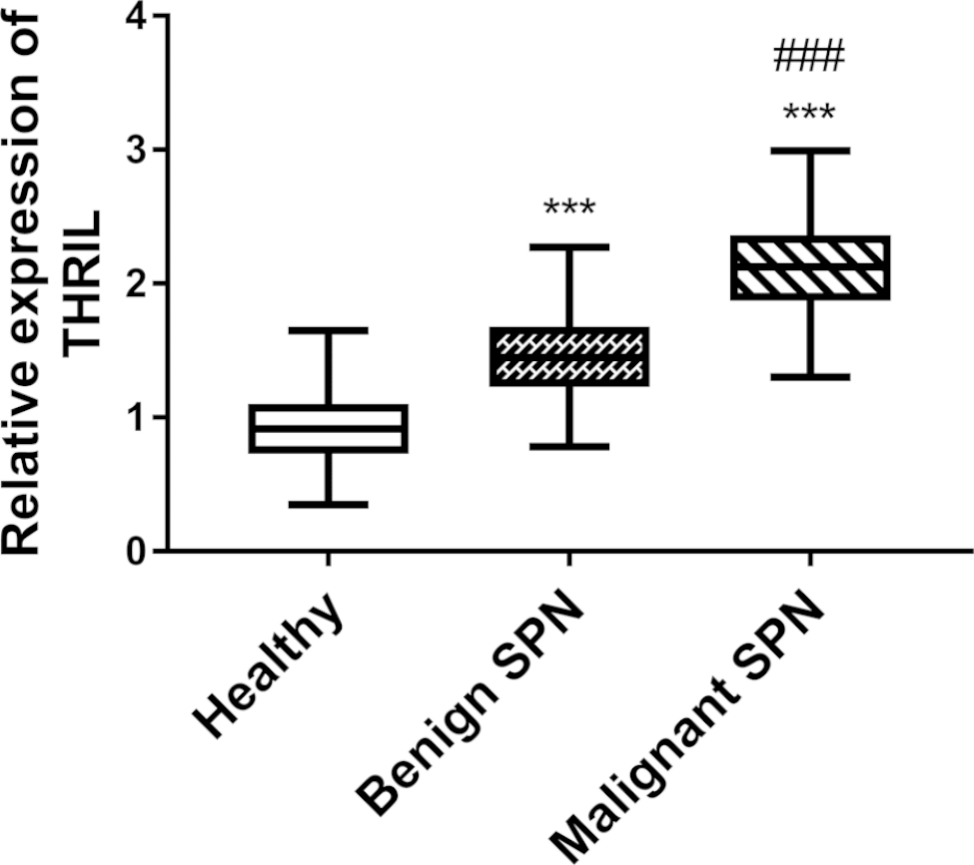
The abundance of THRIL content was observed with the worsening of SPN. ***P < 0.001, relative to healthy group. ###*P* < 0.001, relative to benign SPN group

Through reverse transcription and quantitative PCR analysis, THRIL was performed in patients with malignant and benign SPNs. The expression of THRIL was statistically different among the healthy group, malignant SPN group, and benign SPN group (Fig. 1, P < 0.001). The gradual increase in THRIL levels suggested that SPN progression contributed to the enhanced THRIL content.

### Expression of tumor markers and its interrelationship with THRIL

**Fig. 2:**
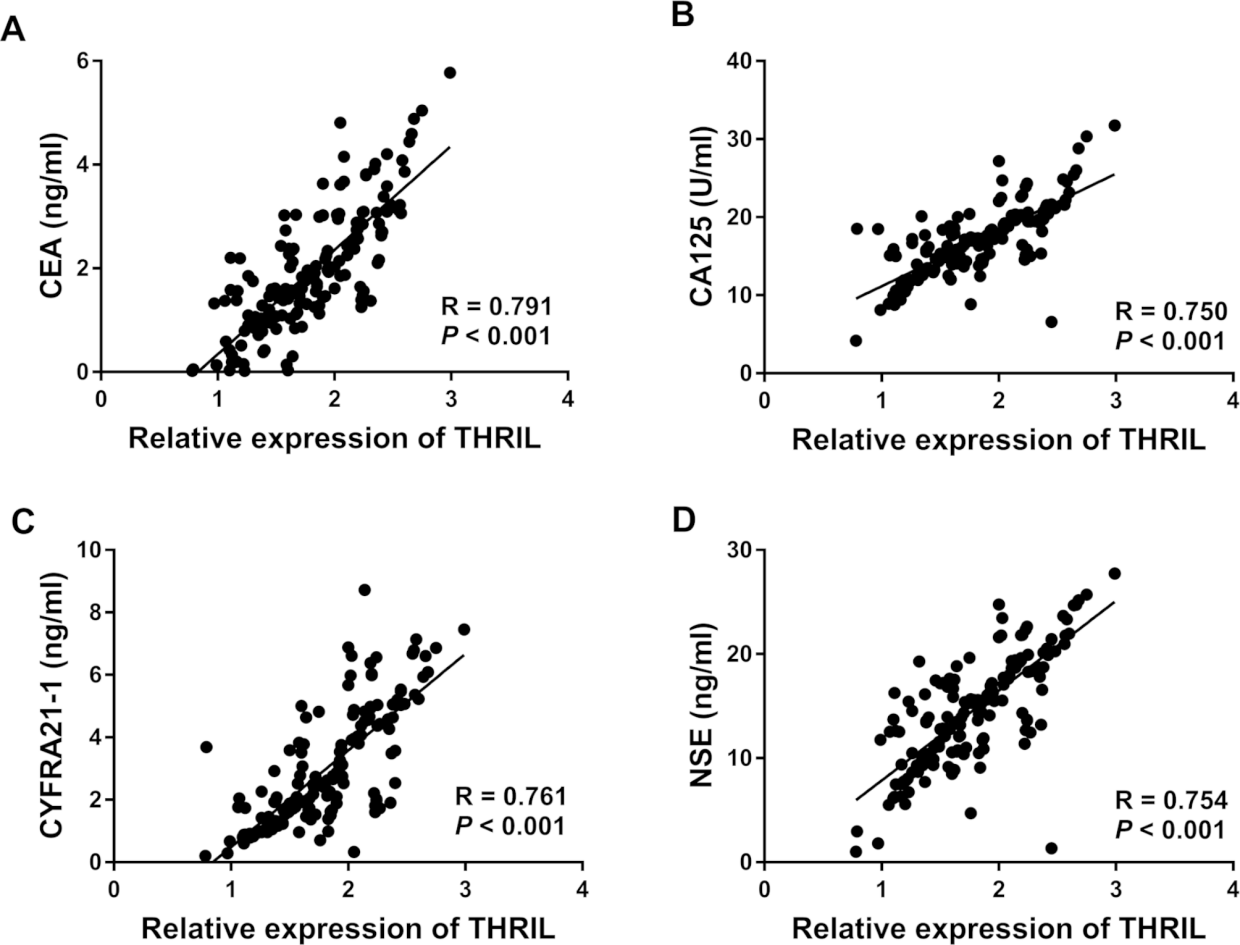
The increase in THRIL was proportional to the elevated levels of (**A**) CEA, (**B**) CA125, (**C**) CYFRA21-1, and (**D**) NSE.

The levels of CYFRA-21 and CEA were raised in the benign SPN group and malignant SPN group relative to the healthy group (Table 3, *P* < 0.001). The concentration of CA125 and NSE in the benign SPN group and malignant SPN group did not differ from those in the healthy group (Table 3, *P* > 0.05). Further analysis documented that THRIL was positively correlated with CEA (Fig. 2A, R = 0.791, *P* < 0.001), CA125 (Fig. 2B, R = 0.750, *P* < 0.001), CYFRA21-1 (Fig. 2C, R = 0.761, *P* < 0.001), and NSE (Fig. 2D, R = 0.754, *P* < 0.001).

**Table 3:** Comparisons of indicators in three groups

Indicators	Healthy group (N = 74)	Benign SPN (N = 83)	Malignant SPN (N = 86)	*P* value
CEA (ng/ml)	1.27 ± 0.47	1.47 ± 0.80	2.41 ± 1.24	< 0.001
CA125 (U/ml)	15.95 ± 2.68	16.43 ± 3.09	17.38 ± 5.25	0.062
CYFRA21-1 (ng/ml)	1.01 ± 0.60	1.71 ± 0.71	4.16 ± 1.78	< 0.001
NSE (ng/ml)	13.50 ± 4.08	14.32 ± 5.10	15.16 ± 5.23	0.102

Acronyms interpretation: CEA, carcinoembryonic antigen; CA125, carbohydrate antigen 125; CYFRA21-1, cytokeratin 19 fragment 21 − 1; NSE, neuron-specific enolase

### Analysis of the efficacy of THRIL in the differential diagnosis of benign and malignant SPNs

**Fig. 3:**
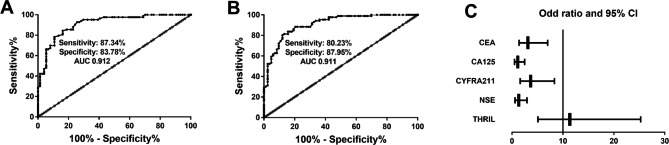
The clinical impact of THRIL in the progression of SPN. (**A**) The ROC curve of THRIL in differentiating benign SPN patients. (**B**) ROC curve confirmed that THRIL could discriminate malignant SPN patients from benign SPN patients. (**C**) Forest plots were drawn to show the odds ratio of multivariate logistic regression

According to the healthy group and benign SPN group, the ROC curve was plotted to evaluate the diagnostic ability of THRIL to distinguish benign SPN. The AUC of the area under the THRIL curve was 0.912 (sensitivity = 87.34%, specificity = 83.78%, Fig. 3A). According to the malignant SPN group and benign SPN group, the ROC curve for malignant SPN screening was plotted. The AUC of the product under the ROC curve was 0.911, the sensitivity was 80.23% and the specificity was 87.95% (Fig. 3B).

The benign SPN and malignant SPN groups were included in this test to analyze the risk of worsening SPN and the results were shown in Table 4. CEA, CYFRA21-1, and THRIL were independent risk factors for predicting malignant SPN and their OR values were 3.118 (95% CI: 1.391–6.989, *P* < 0.01), 3.653 (95% CI: 1.601–8.335, *P* < 0.01), and 11.352 (95% CI: 5.101–25.262, *P* < 0.001), respectively. As documented in Fig. 3C, the OR values for the risk factors were summarized by forest plots.

**Table 4:** Binary regression analysis on variables associated with SPN

Variables	OR	95%CI	*P* value
CEA (ng/ml)	3.118	1.391–6.989	0.006
CA125 (U/ml)	1.123	0.505–2.498	0.776
CYFRA21-1 (ng/ml)	3.653	1.601–8.335	0.002
NSE (ng/ml)	1.323	0.596–2.938	0.492
THRIL	11.352	5.101–25.262	< 0.001

Acronyms interpretation: CEA, carcinoembryonic antigen; CA125, carbohydrate antigen 125; CYFRA21-1, cytokeratin 19 fragment 21 − 1; NSE, neuron-specific enolase

### Effect of silencing THRIL on lung cancer cells

**Fig. 4:**
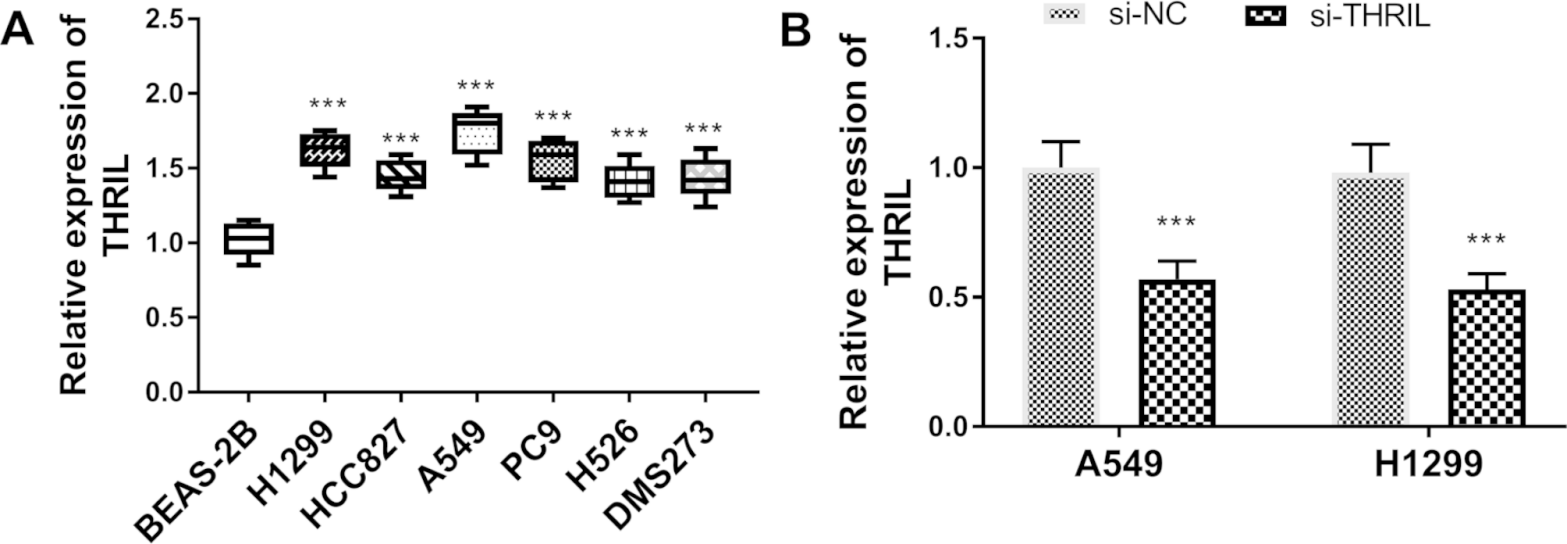
The expression of THRIL in cells. (**A**) An increase of THRIL expression in cancer cell lines compared with BEAS-2B cells. (**B**) In A549 and H1299 cells, THRIL expression was inhibited led by si-THRIL. ***P < 0.001, relative to BEAS-2B or si-NC.

Compared with BEAS-2B, the level of THRIL in lung cancer cells H1299, HCC827, A549, PC9, H526, and DMS273 increased prominently (Fig. 4A, P < 0.001), elucidating abnormal expression of THRIL in lung cancer. Considering the level of THRIL in all lung cancer cell lines of Fig. 4A, A549 and H1299 cells were selected for the follow-up experiment. The si-THRIL was applied to decrease the concentration of THRIL in A549 and H1299 cells (Fig. 4B, P < 0.001).

**Fig. 5:**
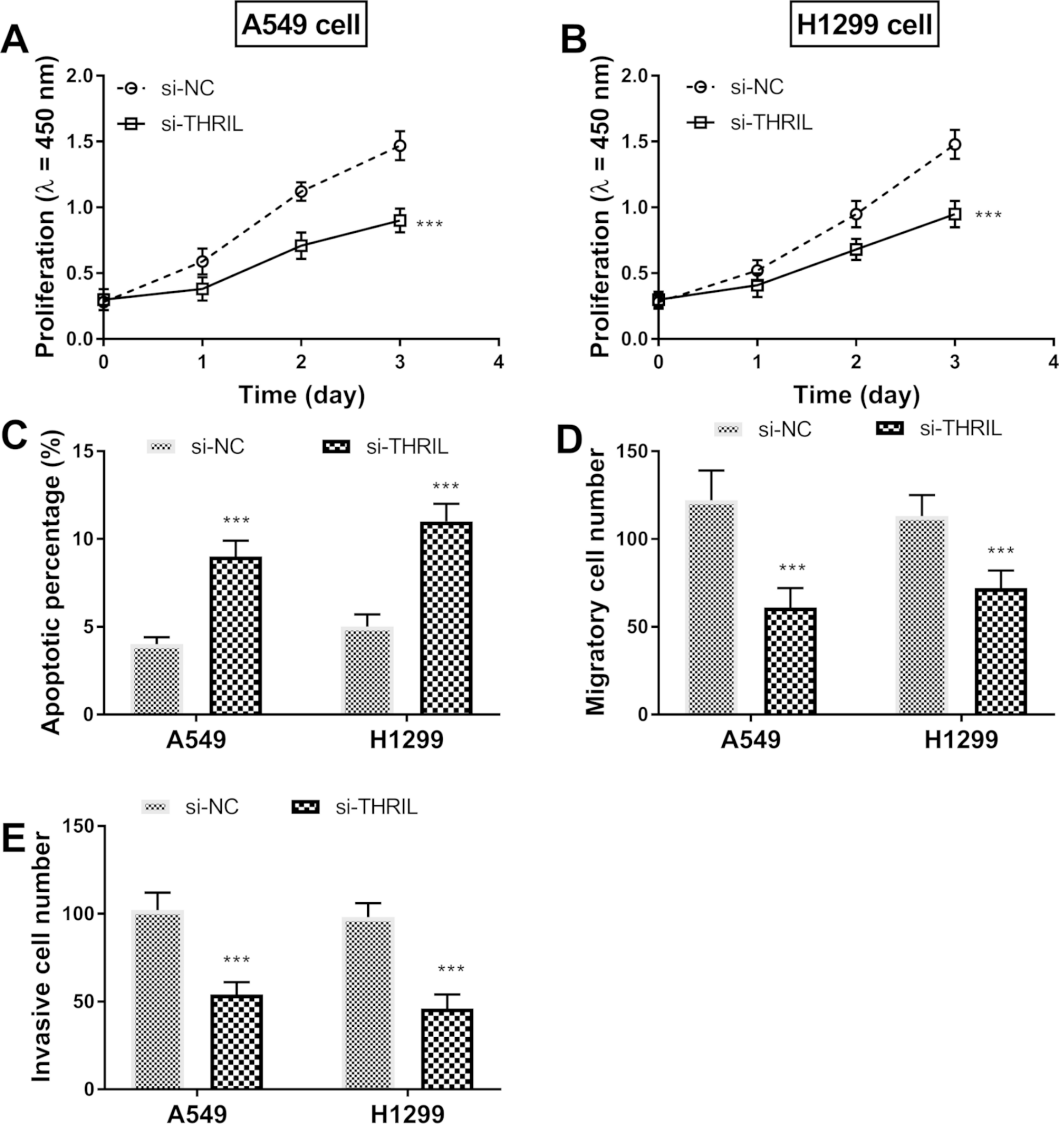
THRIL promotes the activities of lung cancer cells. (**A-B**) The influence of THRIL knockout on the proliferation of A549 and H1299 cells. (**C**) The value of THRIL silencing on apoptosis. (**D-E**) The migration and invasion were inhibited by the interference of THRIL.

The proliferation and apoptosis of A549 and H1299 cells were detected. The survival rate of A549 and H1299 cells of lung cancer in the si-THRIL group decreased (Fig. 5A, B and P < 0.001), supporting that THRIL could accelerate the proliferation of lung cancer cell lines. The results of flow cytometry indicated that the apoptosis rates of lung cancer A549 and H1299 cells in the si-THRIL group were raised (Fig. 5C, P < 0.001). In addition to this, the migration and invasion of cells were also ameliorated because of the silencing THRIL (Fig. 5D, E and P < 0.001).

### THRIL targeting regulates the expression of miR-99a

**Fig. 6:**
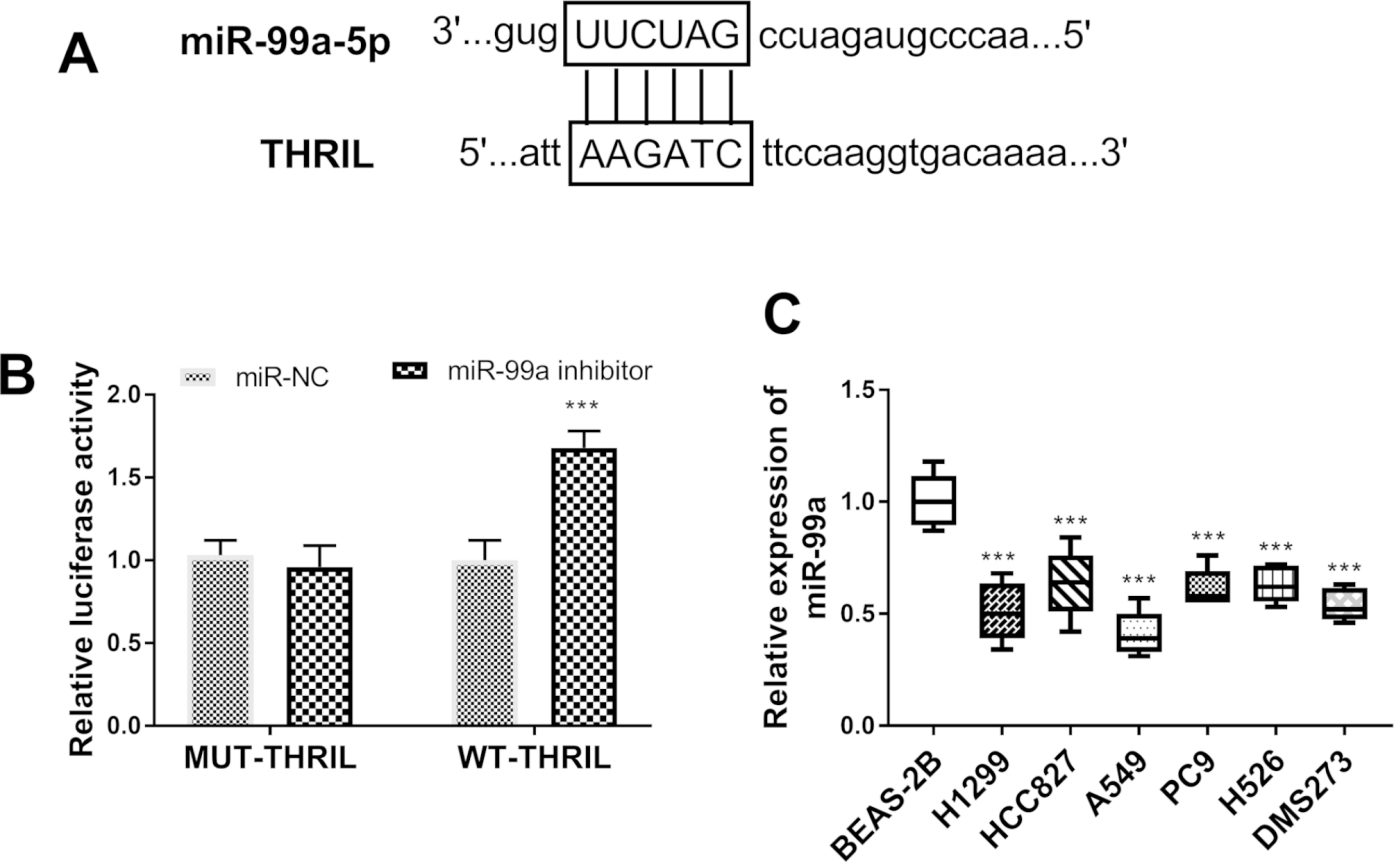
THRIL acts as a molecular sponge of miR-99a. (**A**) Prediction of the binding site of THRIL and miR-99a. (**B**) Luciferase activity transfected with MUT-THRIL or MT-THRIL and miR-99a. (**C**) Expression level of miR-99a lung cancer cell lines (H1299, HCC827, A549, PC9, H526, and DMS273) and normal bronchial epithelial cell line (BEAS-2B). ***P < 0.001, relative to BEAS-2B or si-NC.

The complementary targeting sequences between THRIL and miR-99a were shown in Fig. 6A. The findings of the double luciferase report experiment documented no significant difference in luciferase activity in the THRIL-3’UTR mutant plasmid (Fig. 6B, P > 0.05). MiR-99a can upregulate the activity of the WT-THRIL luciferase reporter gene (Fig. 6B, P < 0.001). We then calculated the concentration of miR-99a in cancer cells, and the results reflected that miR-99a was declined (Fig. 6C, P < 0.001).

### THRIL affects the activities of lung cancer cells by regulating the expression of miR-99a

**Fig. 7:**
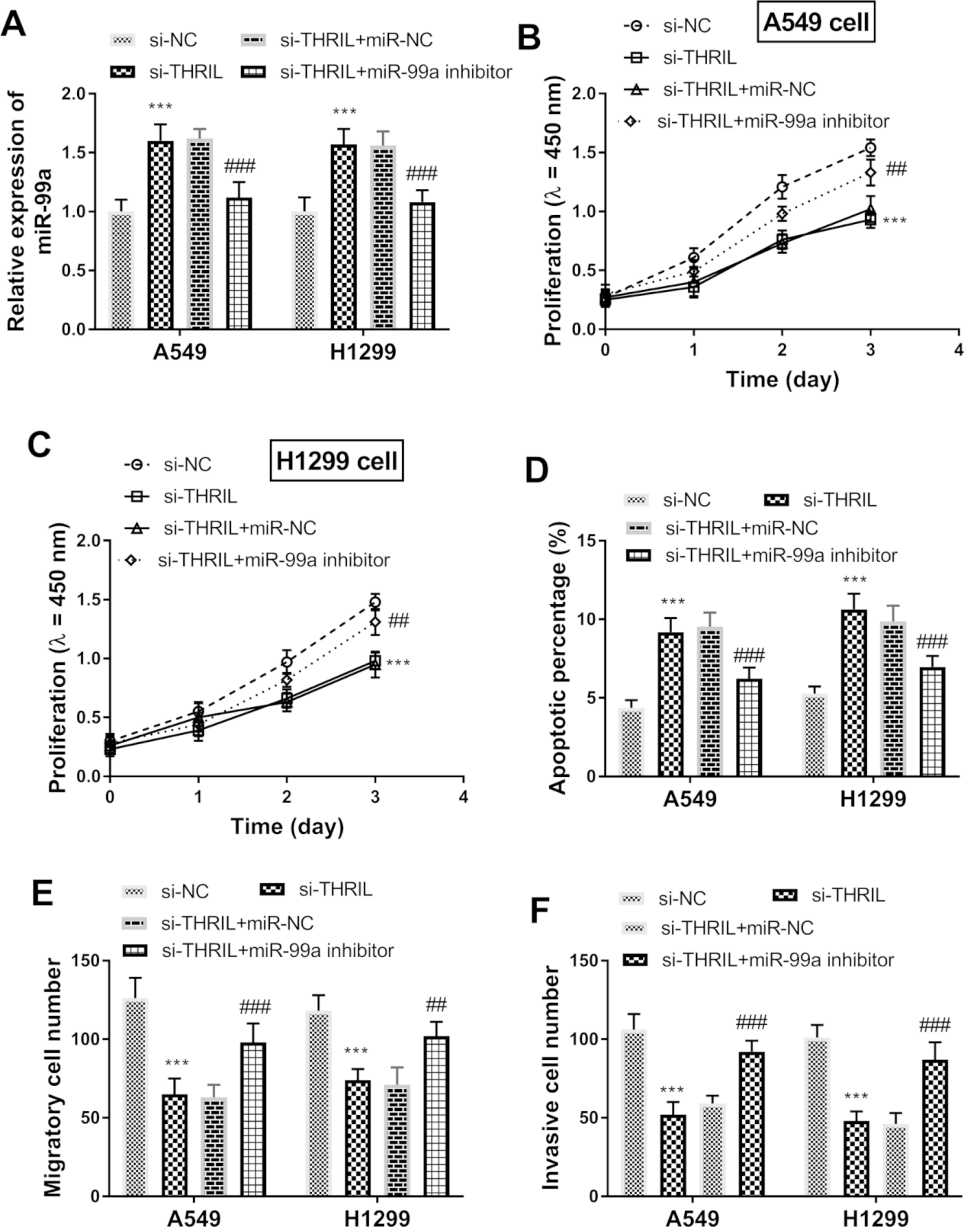
THRIL-mediated miR-99a function in lung cancer cells. (**A**) The expression of miR-99a in A549 and H1299 cells transfected with si-THRIL and THRIL siRNA. (**B-C**) The effect of miR-99a on THRIL-mediated A549 and H1299 cell proliferation. (**D**) The effect of miR-99a inhibitor on si-THRIL-mediated cell apoptosis. (**E**) The impacts of miR-99a on si-THRIL-mediated anti-migration of cells. (**F**) Effects of downregulation of miR-99a expression on invasion of A549 and H1299 cells. ***P < 0.001, relative to si-NC group; ##*P* < 0.01, ###*P* < 0.001, relative to si-THRIL group

RT-qPCR analysis reflected that si-THRIL could upregulate miR-99a levels, while miR-99a inhibitors could inhibit miR-99a levels in A549 and H1299 cells (Fig. 7A, P < 0.001).

To determine whether the carcinogenic effect of THRIL is mediated by miR-99a, we detected the proliferation and apoptosis by CCK-8 and flow cytometry. The miR-99a inhibitor reversed the inhibitory effect of low THRIL expression on cell proliferation (Fig. 7B C, P < 0.01). Silenced miR-99a reversed the pro-apoptotic effects of low THRIL expression (Fig. 7D, P < 0.001). The migratory number and invasive numbers of A549 and H1299 cells were suppressed by the knockdown of THRIL and partially reversed by miR-99a inhibitor (Fig. 7E F, P < 0.001). In conclusion, THRIL improves the proliferation, migration, and apoptosis of lung cancer cells and inhibits apoptosis by down-regulating miR-99a.

### IGF1R is a candidate target of miR-99a

**Fig. 8:**
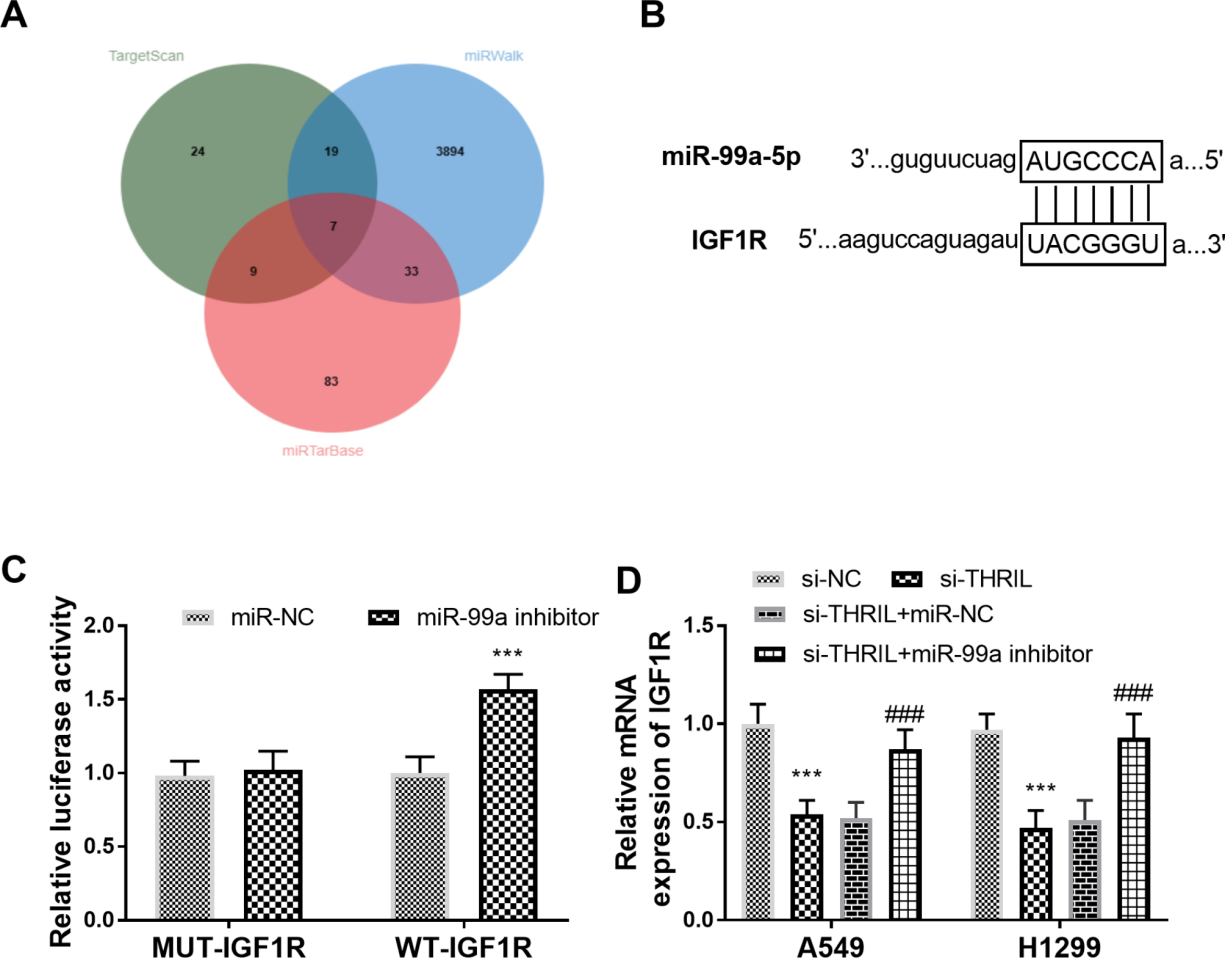
IGF1R is a target of miR-99a. (**A**) The Venn diagram of miR-99a targets. (**B**) The binding site between miR-99a and IGF1R. (**C**) Results of the double luciferase report experiment. (**D**) Relative expression of IGF1R mRNA. ***P < 0.001, relative to si-NC group; ###*P* < 0.001, relative to si-THRIL group

The intersected targets of Venn were shown in Fig. 8A. IGF1R was confirmed as a candidate target gene for miR-99a, and their targeting site was shown in Fig. 8B. After co-transfection of wild-type IGF1R 3’-UTR gene reporter plasmid with miR-99a inhibitors, the luciferase activity was strengthened (Fig. 8C, P < 0.001). The relative expression of IGF1R mRNA in the si-THRIL group was lessened compared with the si-NC group, while that in the si-THRIL + miR-99a inhibitors group was higher than that in the si-THRIL group (Fig. 8D, P < 0.001), indicating that THRIL and miR-99a could work together in the regulation of IGF1R.

## Discussion

Lung cancer is a debilitating disease, and it is one of the most death tumors in the world [20]. The majority of patients are already in the advanced to the advanced stage when they are diagnosed, so timely confirmation is essential to improve the survival outcome of lung tumor patients [21, 22]. With the widespread application of chest CT, more and more SPNs have been diagnosed. Although the detection rate of SPN is increasing, its accurate diagnosis and effective treatment are still hot and difficult points in the medical research field. Timely surgical resection of malignant SPNs has a vital impact on the prognosis of early-stage lung cancer [23]. For SPN with atypical morphological and hemodynamic features, high-resolution CT also has difficulty differentiating benign from malignant [24]. However, existing tumor biomarkers such as carcinoembryonic antigen (CEA) have limited value in the early diagnosis, so the active search for new tumor markers is currently one of the hot spots in clinical research at present.

LncRNAs are widely distributed in eukaryotes and are related to tumors [25]. LncRNAs play a role similar to that of tumor suppressor genes or oncogenes. LncRNA XLOC_009167 is a tool for differentiating patients with lung cancer from patients with pneumonia [16]. LncRNA TUC338 is elevated in lung cancer patients and it has a clinical value [26]. This publication also emphasized that the level of THRIL in serum ascended in benign SPN and malignant SPN groups, indicating the connection between THRIL and SPN. The excessive concentration of THRIL was also manifested in malignant SPN patients in comparison with the benign SPN group, pinpointing that the growth of SPN evokes the increment of THRIL. THRIL is overexpressed in patients with COVID-19 and it can differentiate the acute phase of COVID-19 [27]. In another study of acute lung injury, THRIL is manifested to be upregulated by Chen et al. [18]. Consistent with the previous investigations, THRIL ascended with the SPN progression, suggesting a close correlation between SPN and THRIL.

This study confirmed that the content of CYFRA-21 and CEA were raised in the benign SPN group and malignant SPN group, pinpointing their interconnection with SPN. CEA is an antigen first discovered in colon cancer, and it is a biomarker to aid in the screening of lung cancer [28]. The serum concentration of CEA is an alternative indicator to assess the prognosis of non-small cell lung cancer (NSCLC) [29]. CYFRA21-1 is an epithelial tumor marker involved in common epithelial malignancies, including lung cancer [30]. Pearson correlation analysis manifested that THRIL was linked to the CEA, CA124, CYFRA21-1, and NSE, further supporting the above finding. Specific lncRNAs are potential indicators in the differentiation of patients with lung tumors [31, 32]. The expression of PVT1 and GAC5 is a diacritical sign to distinguish patients with NSCLC [33]. This current project clarified that THRIL could recognize benign SPN from healthy individuals as well as malignant SPN from benign SPN patients, depicting that THRIL might be a risk associated with SPN degeneration and growth. In addition, the regression analysis substantiated that THRIL was implicated in SPN, indicating the correspondence between them.

The function of lncRNA in various tumors, including lung cancer, has attracted increasing attention. THRIL may also participate in cellular activities, as previous studies have shown. Silencing THRIL alters the normal survival activities of fibroblast-like synoviocytes [34]. In retinal microvascular endothelial cells, THRIL exerts stimulatory impacts on cell proliferation and migration [35]. In this study, cell experiments confirmed that the expression of THRIL was significantly upregulated in lung cancer cell lines compared with that in BEAS-2B, which was basically consistent with the previous conclusion of our findings. After the downregulation of THRIL expression, the proliferation, migration, and invasion activities of A549 and H1299 cells were reduced and the apoptosis was raised, indicating that THRIL may play a role in the carcinogenesis and development of lung cancer.

LncRNA competitively binds miRNA and inhibits its function by virtue of miRNA sponge adsorption, thereby affecting the transcription of downstream target mRNA [36]. In this study, miR-99a was found to be a potential target gene of THRIL. The combination of THRIL and miR-99a synergistically regulates the process of myocardial infarction [37]. Yu et al. confirmed that miR-99a is a novel tumor suppressor of NSCLC, which is expressed at low levels in tissues and cell lines [38]. This study found that after down-regulation of THRIL expression in A549 cells, the expression of miR-99a was correspondingly increased. THRIL might reduce its pro-tumor activity by competitively binding to miR-99a, thereby affecting the proliferation, migration, and invasion of lung cancer cells and ameliorating apoptosis. This study also verified that IGF1R was a putative target of miR-99a. The expression of IGF1R, the downstream target gene of miR-99a, was also affected after THRIL silencing. This interaction between IGF1R and miR-99a has been identified in several previous publications, such as cervical cancer, gastric cancer, and esophageal squamous cell carcinoma [39–41]. IGF1R is a typical oncogenic cytokine [42]. IGF1R has been implicated in the chemoradioresistance of colorectal cancer [43]. Many studies have confirmed that IGF1R is highly expressed in lung cancer tissue, and has important impacts on lung cancer. IGF1R expression is related to poor disease-free survival in patients with NSCLC and is a negative factor in disease progression [44]. IGF1R is a pro-cancer indicator in the tumor microenvironment that promotes heterotopic transplantation and initiation [45]. Overexpression of IGF1R contributes to increased survival and inhibited apoptosis of lung cancer cells [46]. Conclusively, IGF1R might participate in the regulation of the THRIL/miR-99a axis in the lung carcinoma cell line.

In summary, the expression of THRIL was increased with the growth of SPN and in line with the increment of tumor markers, such as CEA, CA124, CYFRA21-1, and NSE. The detection of serum THRIL had a high clinical value in the evaluation of benign and malignant SPN patients. In lung cancer cells, THRIL regulated IGF1R through miR-99a and then affected the proliferation, migration, invasion, and apoptosis. THRIL/ miR-99a/IGF1R axis might be a novel potential target in lung cancer.

## Data Availability

The datasets used and/or analysed during the current study are available from the corresponding author on reasonable request.
